# “Homicide and Zonisamide”: a review of the literature and illustrative case report on second and third generation antiseizure medications and psychiatric effects

**DOI:** 10.3389/fpsyt.2026.1805235

**Published:** 2026-06-01

**Authors:** Stefanie Nguyen, Thomas C. Varkey, Briana Khan, M. Christian Cornelius

**Affiliations:** 1 Department of Neurology, The University of Arizona College of Medicine - Phoenix, Phoenix, AZ, United States; 2 Department of Psychiatry, The University of Arizona College of Medicine - Phoenix, Phoenix, AZ, United States

**Keywords:** antiseizure medication (ASM), antiseizure medication side effects, cerebral cavernous malformations, seizures, zonisamide

## Abstract

**Introduction:**

Cerebral cavernous malformations (CCMs) are vascular lesions that can cause focal epilepsy due to local hemosiderin deposition, gliosis, and cortical irritation. Epileptic seizures attributable to CCMs contribute significantly to morbidity and often require long-term management with antiseizure medications (ASMs), but the psychiatric side effect profiles of ASMs remain underreported in the literature.

**Case report:**

Herein, we present a case report of a patient with homicidal ideation following zonisamide use and uptitration following an Epilepsy Monitoring Unit (EMU) stay. Detailed is his hospitalization for this side effect and transition to another ASM so that he could return to a normal life outside the hospital.

**Methods:**

To contextualize this case, a literature review was conducted using PubMed from 1990 to 2026 using combinations of the terms “zonisamide,” “antiepileptic drugs,” “homicidal ideation,” and “psychiatric adverse effects.” Relevant articles were manually reviewed and no prior cases explicitly describing homicidal ideation associated with zonisamide were identified.

**Conclusion:**

Homicidal ideation should be contextualized within a spectrum of ASM-associated psychiatric adverse effects (PAEs). Zonisamide may have contributed to symptom emergence in a susceptible individual within a multifactorial neuropsychiatric context. To our knowledge, this is the first case report explicitly documenting homicidal ideation as an adverse event of zonisamide.

## Introduction

Cerebral cavernous malformations (CCMs) may be diagnosed at any age but are most commonly identified in young to middle adulthood, often following the onset of seizures, focal neurological symptoms, or incidentally during neuroimaging performed for unrelated reasons (1). Diagnosis is primarily established through magnetic resonance imaging (MRI), with susceptibility-weighted imaging (SWI) or gradient-echo sequences demonstrating characteristic “popcorn-like” lesions with a hemosiderin rim (2, 3). Computed tomography (CT) is less sensitive and typically reserved for acute hemorrhage detection (2, 3).

CCMs may occur in sporadic or familial forms, which differ in clinical presentation and diagnostic approach. Sporadic CCMs typically present as a single lesion and are often associated with a developmental venous anomaly, whereas familial CCMs are characterized by multiple lesions, earlier age of onset, and a higher risk of recurrent hemorrhage and seizure recurrence (1, 4). Familial CCMs with pathogenic variants in the *CCM1 (KRIT1), CCM2, or CCM3* genes should be suspected in patients with multiple cavernous malformations, a positive family history, or early-onset disease (1). In such cases, genetic testing is recommended to confirm the diagnosis, guide family counseling, and facilitate screening of at-risk relatives.

Epilepsy is one of the most common clinical manifestations of supratentorial CCMs, occurring in up to 50–70% of symptomatic patients, and is thought to arise from perilesional hemosiderin deposition, gliosis, and chronic cortical irritation (5, 6). While many patients achieve seizure control with antiseizure medications (ASMs), a substantial subset develops drug-resistant epilepsy, often necessitating surgical intervention (7). Patients with familial CCMs frequently undergo repeated neurosurgical procedures and long-term ASM therapy, increasing cumulative neurological and psychiatric morbidity (8).

Second and third generation ASMs are generally favored for improved tolerability and reduced systemic toxicity; however, many side effects have been noted, ranging from weight gain and increases in total cholesterol to more serious metabolic and neuropsychiatric complications (9, 10). Psychiatric adverse effects (PAEs) include depression, anxiety, irritability, aggression, psychosis, and suicidal or homicidal ideation (10, 11). Certain ASMs appear to pose a higher risk for PAEs, particularly in individuals with pre-existing psychiatric illness, structural brain disease, or a history of traumatic or surgical brain injury (8, 11). The phenomenon of “forced normalization,” in which improved seizure control is accompanied by the emergence of severe psychiatric symptoms, further complicates treatment decisions in epilepsy care (12). Additionally, psychiatric comorbidities are highly prevalent among patients with epilepsy, with major depressive disorder and anxiety disorders affecting up to 50% of individuals and contributing significantly to impaired quality of life and increased suicide risk (13). The intersection of epilepsy, structural brain pathology, genetic vulnerability, and ASM-related PAEs presents a complex clinical challenge requiring careful medication selection and multidisciplinary management.

Herein, we provide a narrative review of the existing literature on psychiatric effects of second and third generation ASMs and an illustrative case report of a 25-year-old male with a genetically confirmed CCM1 mutation, history of multiple craniotomies for cavernous malformation resection, refractory seizure disorder, major depressive disorder, unspecified anxiety disorder, and prior suicide attempts, who presented to the Emergency Department with acute homicidal ideation temporally associated with initiation of a third-generation ASM. This case underscores the importance of educating patients about rare but serious adverse effects of ASMs, especially in high-risk patients, and it highlights the need for vigilant psychiatric monitoring in the management of CCM-associated epilepsy.

## Case presentation

The patient is a 25-year-old male with a family history significant for psychiatric disorders and a medical history of cavernous vein malformation with confirmed CCM1 gene, status post 3 craniotomies, seizure disorder, major depressive disorder, unspecified anxiety disorder, and prior suicide attempts who presented to the Emergency Department for homicidal ideation toward his father. He reported a history of these intrusive homicidal thoughts for approximately two years, with worsening severity over time and increasing interpersonal conflict with his parents. Days after being kicked out of his parents’ home, he experienced a dissociative episode with visual hallucinations of blood and body parts, and a strong desire to chop his father into pieces, then use hydrogen peroxide or sulfuric acid to burn the body parts and hide the blood. He recognized this was not normal and voluntarily sought emergency hospital evaluation. He reported a temporal relationship between symptom onset and initiation of zonisamide approximately two years prior. He described a reproducible pattern in which homicidal ideation emerged daily after medication administration at approximately 09:00, peaking around 12:00 (approximately 3 hours post-dose), and gradually subsiding thereafter. He denied current suicidal ideation but endorsed chronic depressive symptoms including anhedonia, fatigue, insomnia, psychomotor slowing, feelings of worthlessness, emotional lability, unstable interpersonal relationships, chronic emptiness, and unstable self-image. He denied active intoxication at presentation. Alcohol use was reported as intermittent, ranging from 1–2 beers several times per month to episodic heavy use up to one-fifth of liquor daily. No acute withdrawal symptoms were noted at presentation.

On physical examination, the patient was afebrile and hemodynamically stable. General examination was otherwise unremarkable, with no signs of acute distress, intoxication, or systemic illness. On the mental status exam, the patient was awake, alert, and oriented to time, place, person, and situation. Speech was coherent and goal-directed. Attention and concentration were intact. Remote and recent memories were preserved. Thought process was linear, though thought content was notable for prior intrusive violent ideation as described above. He demonstrated good insight into the abnormality of his thoughts at the time of evaluation and demonstrated intact judgment during hospitalization. Neurologic examination was significant for chronic left-sided upper motor neuron deficits, including:

- Decreased strength (4/5) in the left upper and lower extremities compared to 5/5 on the right
- Increased tone with spasticity on the left side
- Hyperreflexia involving the left upper and lower extremities
- Sensory reduction throughout the left hemibody
- Positive Babinski and Hoffman signs on the left

These findings were consistent with chronic structural injury related to prior CCM-related hemorrhage and surgical interventions.

## Differential diagnosis

The patient’s presentation was initially considered within a broad differential diagnosis, including:

- Primary psychotic disorder
- Substance-induced psychotic or mood disorder
- Mood disorder with psychotic features
- Delirium or metabolic encephalopathy
- Seizure-related behavioral disturbance or postictal psychiatric phenomena
- Medication-induced PAE

Given the complexity of his neuropsychiatric history and structural brain disease, diagnostic evaluation focused on excluding reversible metabolic, toxicologic, and acute neurologic causes of behavioral dysregulation.

## Initial labs and studies

During initial evaluation in the emergency department, laboratory studies—including complete blood count, comprehensive metabolic panel, thyroid-stimulating hormone, acetaminophen level, salicylate level, plasma ethanol level, and urine drug screen—were all within normal limits.

Upon admission, additional investigations were obtained, including serum zonisamide level, magnesium, vitamin B12, folate, vitamin D (25-OH), rapid plasma reagin, and hemoglobin A1C, all of which were within normal limits. An electrocardiogram demonstrated no abnormalities. A spot electroencephalogram showed evidence of right hemispheric cortical dysfunction, consistent with prior structural injury, without epileptiform discharges or electrographic seizures. Prior brain MRI was reviewed (**Figure 1**) and showed no acute changes. The patient’s serum zonisamide level was within the therapeutic range (31.3 mcg/mL).

**Figure 1:**
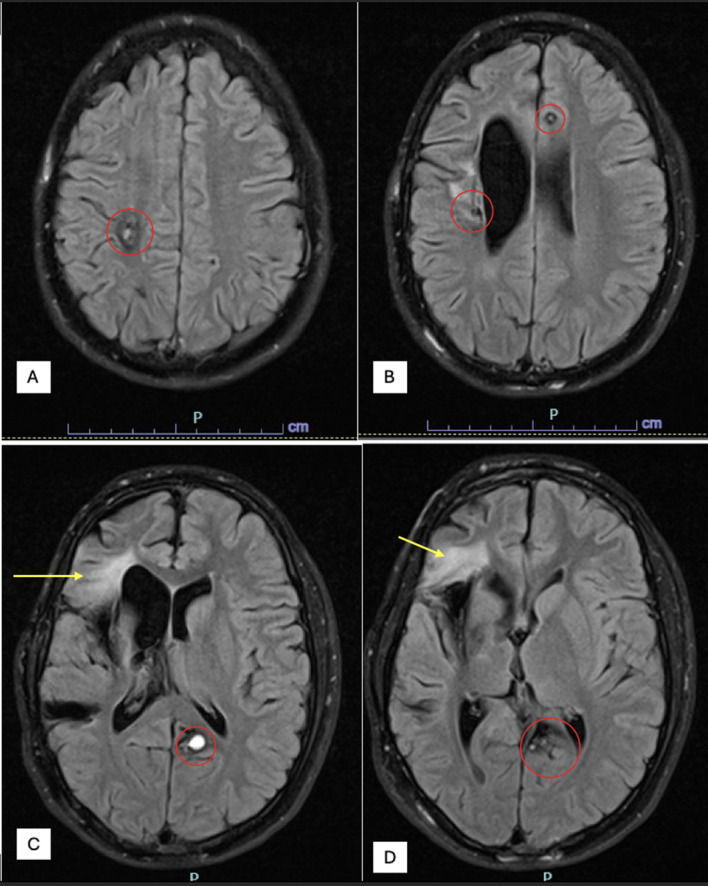
T2 Fluid-Attenuated Inversion Recovery (FLAIR) MRI Brain showing cavernous malformation “popcorn” lesions best seen on images A, B, and C within the red circles. Areas of gliosis seen in the right frontal lobe on images C and D with yellow arrows pointing at the gliosis.

Based on the absence of metabolic, toxicologic, or acute structural abnormalities, alternative diagnoses, including primary psychotic disorder, substance-induced psychosis, delirium, and acute neurologic pathology, were considered less likely. Notably, the patient reported that his homicidal ideation began after initiation of zonisamide and demonstrated a consistent temporal pattern, with peak intensity occurring approximately three hours after dosing. This temporal association, along with subsequent symptom resolution following discontinuation of zonisamide, suggests a possible medication-related contribution within a multifactorial clinical context.

## Clinical management

The patient was admitted to inpatient behavioral health for safety, diagnostic clarification, and medication management. Given the concern for medication-associated PAE, a structured pharmacologic intervention was initiated. He was placed on seizure precautions with plans to cross-titrate the zonisamide with a loading dose of lacosamide pending initial workup. The decision to discontinue zonisamide was based on concern for a potential PAE in a high-risk patient with pre-existing psychiatric illness and structural brain disease. Alternative antiseizure medications were carefully considered. Valproate (divalproex) was avoided due to its known association with thrombocytopenia and platelet dysfunction, which may increase bleeding risk, particularly in patients with underlying vascular malformations such as cerebral cavernous malformations (CCMs). Lamotrigine was also considered given its favorable neuropsychiatric profile and mood-stabilizing properties. However, the patient had previously discontinued lamotrigine due to reported adverse effects, and re-initiation was deferred in favor of an alternative agent. Lacosamide was ultimately selected due to its relatively favorable neuropsychiatric tolerability, minimal drug–drug interactions, and efficacy in focal epilepsy. A cross-titration strategy was employed to minimize the risk of breakthrough seizures during the transition. The patient’s existing psychiatric medication regimen was also reviewed. Trazodone was discontinued due to its potential to lower seizure threshold, particularly in the context of active medication adjustments and increased seizure vulnerability. Duloxetine was continued to maintain baseline management of depressive and anxiety symptoms, as the patient had previously tolerated this medication without reported adverse effects. Non-pharmacologic interventions included close psychiatric monitoring, safety precautions, and supportive therapy focused on distress tolerance and reality testing. The patient was maintained in a structured inpatient setting to ensure safety given the severity of presenting symptoms.

During the inpatient hospitalization, the patient was closely monitored for both neuropsychiatric symptoms and seizure activity following discontinuation of zonisamide and initiation of cross-titration to lacosamide. Clinician-assessed outcomes included serial mental status examinations, behavioral observations, and neurologic assessments.

During hospitalization, the patient experienced an increase in seizure aura frequency following zonisamide discontinuation. In response, lorazepam 1 mg three times daily was initiated as a temporary adjunctive measure to reduce the risk of breakthrough seizures during the transition period. This intervention was intended as a short-term strategy pending stabilization on lacosamide.

Within several days of zonisamide discontinuation, the patient demonstrated complete resolution of homicidal ideation, with no recurrence throughout the remainder of hospitalization. He remained free of psychotic symptoms, and no further dissociative episodes or perceptual disturbances were observed. He was subsequently discharged to a residential treatment facility for continued psychiatric stabilization and medication management. Per available records, there was no recurrence of homicidal ideation following sustained discontinuation of zonisamide. The patient reported his incredible gratitude for the care provided the last time he was seen by the team and has been demonstrating better more stable mood since discharge. (Please see **Table 1** for a detailed clinical history as detailed in the text above).

**Table 1:** Clinical timeline.

Baseline: Prior to Zonisamide	CCM1 mutation Multiple craniotomies Refractory epilepsy Major depressive disorder Anxiety disorder Prior suicide attempts
2 years prior to ED presentation: Initiation of zonisamide	Zonisamide 100mg PO QD started for seizure management
Weeks – months after initiation of zonisamide	Emergence of intrusive thoughts toward father
Ongoing symptom pattern	Symptoms peak about 3 hours after each zonisamide dose
Day 0 (ED presentation): Symptom escalation with hospital admission	Dissociative episode with violent visual hallucinations and detailed homicidal ideation
Day 0–1 Initial workup	Labs: CBC, CMP, TSH, Toxicology, Zonisamide level, Serum Magnesium, Vitamin B12 level, Folate level, Vitamin D-OH25, Rapid Plasma Reagin, Hemoglobin A1C Diagnostics: EKG, EEG Imaging: Prior MRI reviewed Findings: no acute metabolic or structural cause identified
Hospital Course	zonisamide tapered lacosamide initiated trazodone discontinued duloxetine continued
Hospital complications: day 11	Breakthrough seizure with aura Lorazepam 1mg TID initiated
Patient stabilization and discharge day 21	ASM regimen: Lacosamide 200mg PO BID + Lorazepam 1 mg PO PRN for seizure aura

## Discussion

This case highlights the complex interplay between structural brain disease, genetic vulnerability, psychiatric comorbidity, and ASM–related PAEs. The presentation of acute homicidal ideation temporally associated with zonisamide initiation underscores the importance of recognizing rare but serious PAEs in patients with epilepsy, particularly those with pre-existing mental illness and underlying cerebral pathology. However, the findings should be interpreted within a multifactorial framework, and causal inference cannot be definitively established.

Psychiatric comorbidity is highly prevalent among patients with epilepsy, affecting up to half of this population, and is independently associated with increased mortality and violence risk (13). In such patients, ASMs may exacerbate pre-existing psychopathology or unmask latent psychiatric or behavioral dysregulation rather than serve as the sole etiologic factor (11). Epilepsy associated with CCMs is a well-established clinical entity, especially in patients with supratentorial lesions and familial disease due to *CCM1* mutations (4, 14). These patients frequently experience refractory seizures, often requiring multiple neurosurgical interventions (7, 8). Recurrent hemorrhage, gliosis, and chronic cortical disruption are increasingly recognized as contributors not only to epilepsy but also to long-term neuropsychiatric morbidity, including mood instability, impulsivity, and affective dysregulation (13). Importantly, these risks exist independent of ASM exposure, complicating attribution when psychiatric symptoms emerge during treatment.

Recent large-scale cohort studies and systematic reviews demonstrate that while PAEs are a class-wide phenomenon among ASMs, the magnitude and type of psychiatric dysregulation vary significantly across agents, with certain medications conferring disproportionately higher risk in patients with underlying psychiatric vulnerability (15–21). Second and third generation ASMs, including zonisamide, are commonly selected for their favorable seizure control and tolerability profiles; however, accumulating evidence suggests that they may provoke significant behavioral and psychiatric side effects in susceptible individuals (11, 22). Agents such as perampanel and levetiracetam have higher reported rates of aggression and irritability, whereas lamotrigine and valproate may exert mood-stabilizing effects and are preferentially selected in patients with comorbid affective disorders (16, 23). Zonisamide occupies an intermediate position within this spectrum; while not among the highest-risk agents, it has been associated with depression, anxiety, agitation, and psychosis, particularly in susceptible individuals (24). Of note, population-based studies suggest that severe PAEs with zonisamide remain uncommon overall, indicating that such reactions may occur primarily in susceptible individuals (25). Proposed mechanisms underlying the behavioral and psychiatric effects of ASMs include GABAergic modulation, glutamatergic inhibition, carbonic anhydrase inhibition, and downstream effects on serotonergic and dopaminergic pathways within frontolimbic circuits (19). **Table 2** lists the most common adverse reactions of second and third generation ASMs reported in the FDA Drug Label, as well as PAEs reported by the FDA and in the current literature. Perampanel is the only third-generation ASM with documented reports of homicidal ideation in its FDA label or published literature to our knowledge (26).

**Table 2:** Second and third generation anti-seizure medications and their side effects.

Year of market release (10)	Medication	FDA label most common adverse reactions in adults	Psychiatric side effects (11, 27) % incidence in parentheses when available
2000	Levetiracetam^A^	somnolence, asthenia, infection, dizziness (28)	suicidal behavior/ideation (0.2%), psychosis (0.6-1.3%), anxiety (1.9-2.5%), depression (4-7.3%), irritability (9-12.5%), anger (2.5%), aggression (1.4-2.6%), behavioral change (3.5%) (15, 29, 30)
2000	Zonisamide^B^	anorexia, dizziness, ataxia, agitation/irritability, memory/concentration difficulty (31)	psychosis (0.8-1.6%), anxiety (1.3-1.6%), depression (4.2-4.3%), irritability (2.6-3.2%), aggression (0.4%), behavioral change (0.5%) (29–31)
2004	Pregabalin^C^	dizziness, somnolence, dry mouth, edema, blurred vision, weight gain, abnormal thinking (concentration difficulty) (32)	suicidal behavior/ideation, insomnia, confusion, attention disturbance, anxiety, depression, euphoric mood; withdrawal related psychiatric symptoms reported (11, 27, 32)
2004	Fosphenytoin^D^	pruritus, nystagmus, dizziness, somnolence, ataxia (33)	psychosis, amnesia, depersonalization, personality disorder, insomnia, confusion, agitation, depression, hostility, emotional lability (11, 27, 33)
2007	Lacosamide^D^	diplopia, headache, dizziness, nausea, somnolence (34)	suicidal behavior/ideation, psychotic disorder, hallucinations, mood alteration, insomnia, confusion, agitation, aggression, depressed mood (11, 27, 34)
2008	Rufinamide^D^	headache, dizziness, fatigue, somnolence, nausea (35)	suicidal behavior/ideation, anxiety (11, 27, 35)
2009	Eslicarbazepine^E^	dizziness, somnolence, nausea, headache, diplopia, vomiting, fatigue, vertigo, ataxia, blurred vision, tremor (36)	suicidal behavior/ideation, insomnia, depression (11, 27, 36)
2012	Perampanel^F^	dizziness, somnolence, fatigue, irritability, falls, nausea, weight gain, vertigo, ataxia, headache, vomiting, contusion, abdominal pain, anxiety (37)	suicidal behavior/ideation, homicidal ideation, psychotic disorder, abnormal behavior, euphoric mood, mood alteration, hypersomnia confusion, anxiety, irritability (12.3%), agitation, aggression (4.4%), anger (2.5%) (11, 15, 27, 37)
2016	Brivaracetam^G^	somnolence, dizziness, fatigue, nausea/vomiting (38)	suicidal behavior/ideation, psychosis, psychotic disorder, hallucination, paranoia, psychomotor hyperactivity, abnormal behavior, adjustment disorder, affect lability, mood alteration, anxiety, irritability (5.6%), aggression (2.5%), anger (3.3%), depression (11, 15, 27, 38)
2018	Stiripentol^D^	somnolence, decreased appetite, weight loss, agitation, ataxia, hypotonia, nausea, tremor, dysarthria, insomnia (39)	suicidal behavior/ideation, insomnia, agitation, aggression (11, 27, 39)
2018	Cannabidiol^D^	somnolence, decreased appetite, diarrhea, transaminase elevations, fatigue, rash, sleep disturbance, infections (40)	suicidal behavior/ideation, sleep disorder, insomnia, anxiety, irritability, agitation, aggression (11, 27, 40)
2018	Everolimus^H^	stomatitis, sinusitis, otitis media, infections/upper respiratory tract infection, asthenia, pyrexia, fatigue, cough, diarrhea (41)	insomnia (9%) (41)
2019	Cenobamate^D^	somnolence, dizziness, fatigue, diplopia, headache (42)	suicidal behavior/ideation, euphoric mood, confusion, irritability (11, 27, 42)
2020	Fenfluramine^D^	somnolence, fatigue, ataxia, fall, increased blood pressure, drooling/salivary hypersecretion, pyrexia, upper respiratory tract infection, diarrhea, constipation, vomiting, decreased appetite, weight loss, abnormal echocardiogram, status epilepticus (43)	suicidal behavior/ideation, insomnia, mood swings, anxiety, restlessness, agitation, irritability, anger (11, 27, 43)

A-H Definitions (FDA incidence thresholds).

A = ≥ 5% more than placebo.

B = ≥ 4% more than placebo.

C = ≥ 5% and >2x placebo.

D = ≥ 10% and more than placebo.

E = ≥ 4% and ≥2% more than placebo.

F = ≥ 5% and ≥1% more than placebo.

G = ≥ 5% and ≥2% more than placebo.

H = ≥ 30%.

In this case, alternative etiologies—including primary psychotic disorders, substance-induced states, metabolic abnormalities, and acute intracranial pathology—were systematically considered and excluded through clinical assessment and diagnostic testing. The temporal and pharmacokinetic correlation is consistent with prior reports describing severe PAEs emerging shortly after ASM initiation or dose escalation (18, 19). The reported pharmacokinetic peak of zonisamide (approximately 2–5 hours post-dose) is temporally consistent with the patient’s described symptom peak, although interindividual variability limits direct inference (44). A formal causality assessment using the Naranjo scale yielded a score of 6, indicating a *probable* adverse drug reaction. This was based on temporal association, improvement upon discontinuation, dose-responsive relationship, and absence of alternative causes. Despite this, causality cannot be definitively established given multiple confounding factors, including major depressive disorder, prior suicidality, structural brain disease, and prior neurosurgical interventions. These factors independently increase the risk for psychiatric dysregulation and limit causal attribution. As such, the patient’s presentation is best understood as multifactorial, with zonisamide representing a potential precipitating or exacerbating factor within a broader neuropsychiatric vulnerability framework rather than as a sole etiologic agent.

The phenomenon of forced normalization, in which improved seizure control is paradoxically associated with the emergence of psychiatric symptoms, was also considered (12). Although the temporal relationship raises this possibility, the absence of concurrent seizure documentation at the time of symptom emergence limits definitive application of this concept in the present case.

This report has several limitations inherent to its design. As a single-case observation, it is subject to selection bias, recall bias, and lack of generalizability. The absence of rechallenge precludes stronger causal inference. Additionally, the multifactorial nature of the patient’s neuropsychiatric condition limits attribution of symptoms to a single pharmacologic agent. Nevertheless, strengths of this report include detailed temporal characterization of symptom onset relative to medication exposure, comprehensive exclusion of alternative medical causes through multimodal diagnostic evaluation, and structured application of an adverse drug reaction causality assessment tool.

This case illustrates a possible association between zonisamide exposure and severe psychiatric dysregulation in a patient with significant neurological and psychiatric vulnerability. ASM-associated PAE ranges from irritability and aggression to psychosis and suicidal or homicidal ideation. These manifestations are not mutually exclusive and likely reflect shared underlying neurobiological mechanisms. While causality cannot be established, the temporal relationship and clinical course highlight the importance of heightened vigilance for neuropsychiatric adverse effects of antiseizure medications in susceptible individuals. Current guidelines emphasize the importance of counseling patients regarding serious adverse events, even when incidence is low, and recommend multidisciplinary collaboration when behavioral symptoms arise during epilepsy treatment (45). In select patients, early consideration of surgical management or alternative ASMs with lower psychiatric risk profiles may reduce morbidity (7, 45). Further systematic studies are needed to better characterize risk factors for severe PAEs across ASM classes.

## Conclusion

This case highlights a possible association between zonisamide and severe PAEs, including homicidal ideation, in a high-risk patient. While a causal relationship cannot be established, the clinical course raises the possibility that zonisamide may contribute to neuropsychiatric symptom exacerbation in vulnerable individuals. These findings should be interpreted cautiously in the context of substantial confounding factors inherent to structural brain disease and pre-existing psychiatric illness.

This report underscores the importance of continued vigilance for PAEs associated with ASMs, particularly in high-risk populations. Clinicians should maintain a high index of suspicion for medication-induced behavioral changes in high-risk populations, as early recognition and intervention may prevent catastrophic outcomes.

## Data Availability

The original contributions presented in the study are included in the article/supplementary material. Further inquiries can be directed to the corresponding author.
